# Supplementary material for: Targeting NLRP3 signaling with a novel sulfonylurea compound for the treatment of vascular cognitive impairment and dementia
Source: Fluids Barriers CNS. 2025 Jun 3;22:55. doi: 10.1186/s12987-025-00665-6 (PMC12131594; doi:10.1186/s12987-025-00665-6)

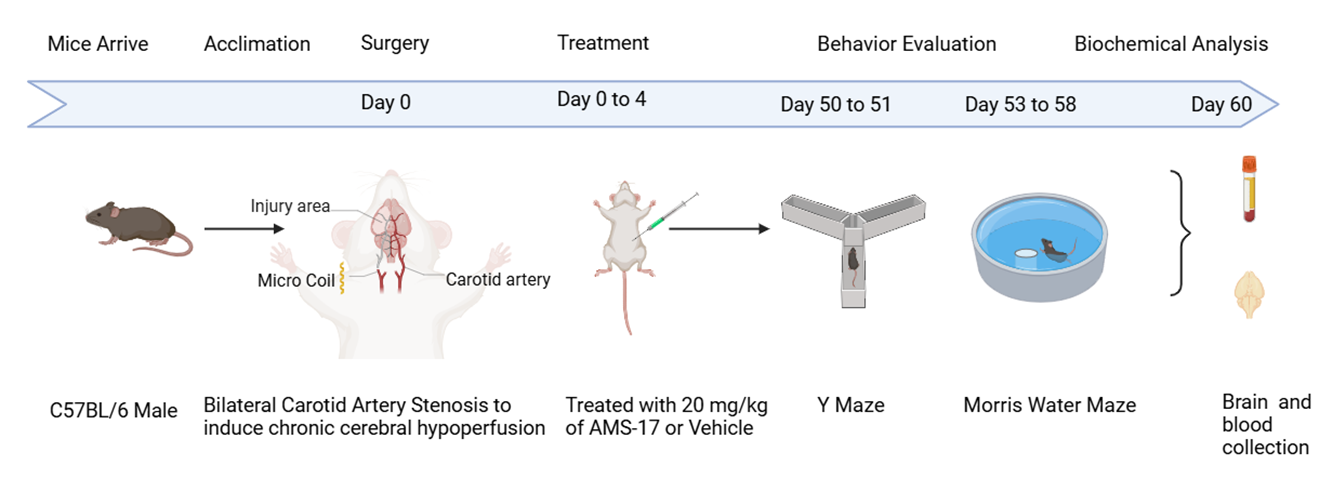


**Supplementary Figure 1.** Experimental outline for the evaluation of the therapeutic effects of AMS-17 in a VaD mouse model (Created with BioRender.com).

**Tables**

| Antibody | Host | Product details | Dilution |
| --- | --- | --- | --- |
| Fibrinogen | Mouse | Proteintech, Cat # 66158-1 | 1:100 |
| CD31 | Rat | BD Pharmingen, Cat # 55027 | 1:20 |
| Occludin | Mouse | Proteintech, Cat # 66378-1 | 1:50 |
| Claudin 5 | Rabbit | Invitrogen, Cat # 34-1600 | 1:50 |
| MBP | Rat | Novus, Cat #NB600-717 | 1:100 |
| NeuN | Rabbit | Cell Signaling, Cat # 24307S | 1:100 |
| CD68 | Mouse | Invitrogen, Cat # MA5-16674 | 1:50 |
| NLRP3 | Rabbit | Novus, Cat #NBP2-12446 | 1:50 |
| NeuN | Mouse | Millipore Sigma, Cat #MAB377 | 1:50 |
| ASC | Rabbit | Cell Signaling, Cat # 67824 | 1:100 |
| P-MST1 (Thr183) | Rabbit | Invitrogen Cat # PA5-4017**7** | 1:10 |
| NG-2 | Rabbit | Abcam Cat # ab275024 | 1:50 |

**Supplementary Table S1 List of primary antibodies used in immunostaining assays**

| Antibody | Host | Product details | Dilution |
| --- | --- | --- | --- |
| GAPDH | Rabbit | Cell Signaling, Cat # 2118 | 1:1000 |
| NLRP3 | Rabbit | Cell Signaling, Cat #15101 | 1:1000 |
| Occludin | Rabbit | Invitrogen, Cat # 40-4700 | 1:1000 |
| Claudin 5 | Rabbit | Invitrogen, Cat # 34-1600 | 1:1000 |
| ASC | Rabbit | Cell Signaling, Cat # 67824 | 1:1000 |
| HSP90 | Rabbit | Cell Signaling, Cat # 4874 | 1:1000 |
| Pro-caspase-1 & cleaved caspase-1 | Rabbit | Proteintech, Cat # 22915-1-AP | 1:1000 |
| P-MST1 (Thr183) | Rabbit | Invitrogen, Cat # PA5-4017**7** | 1:1000 |
| MST1 | Rabbit | Cell Signaling, Cat # 3682 | 1:1000 |

**Supplementary Table S2 List of Western blotting primary antibodies**

| Antibody | Anti | Product details | Dilution |
| --- | --- | --- | --- |
| Alexa Fluor^®^ 488 | Rabbit | Cell Signaling, Cat # 4412 | 1:1000 |
| Alexa Fluor^®^ 555 | Rabbit | Cell Signaling, Cat # 4413 | 1:1000 |
| Alexa Fluor^®^ 555 | Mouse | Cell Signaling, Cat # 4409 | 1:1000 |
| Alexa Fluor^®^ 488 | Mouse | Cell Signaling, Cat # 4408 | 1:1000 |
| Alexa Fluor^®^ 488 | Rat | Cell Signaling, Cat # 4417 | 1:1000 |
| Alexa Fluor^®^ 555 | Rat | Cell Signaling, Cat # 4418 | 1:1000 |

**Supplementary Table S3 List of secondary antibodies used in immunostaining assays**

| **Cytokines** | **Product Details** | **Limit of detection** | **Range of detection** |
| --- | --- | --- | --- |
| **TNF-α** | R&D Systems, Cat # MTA00B-1 | 7.21 pg/mL | 10.9 - 700 pg/mL |
| **IL-1 beta** | Invitrogen, Cat # BMS6002-2 | 1.2 pg/mL | 7.8-500 pg/mL |
| **IL-4** | Invitrogen, Cat # BMS613 | 2.0 pg/mL | 3.9-250 pg/mL |
| **AST** | Abcam, Cat # ab263882 | 39 pg/mL | 125 – 8000 pg/mL |
| **ALT** | Abcam, Cat # ab282882 | 4 pg/mL | 46.88 - 3000 pg/mL |
| **BUN** | Invitrogen, Cat # EIABUN | 0.030 mg/dL | 0.156–10 mg/dL |
| **Creatinine** | Invitrogen, Cat # EIASCR | 0.081 μg/dL | 0.5–4 mg/dL |

**Supplementary Table S4 List of ELISA kits with Limit and Range of detection.**


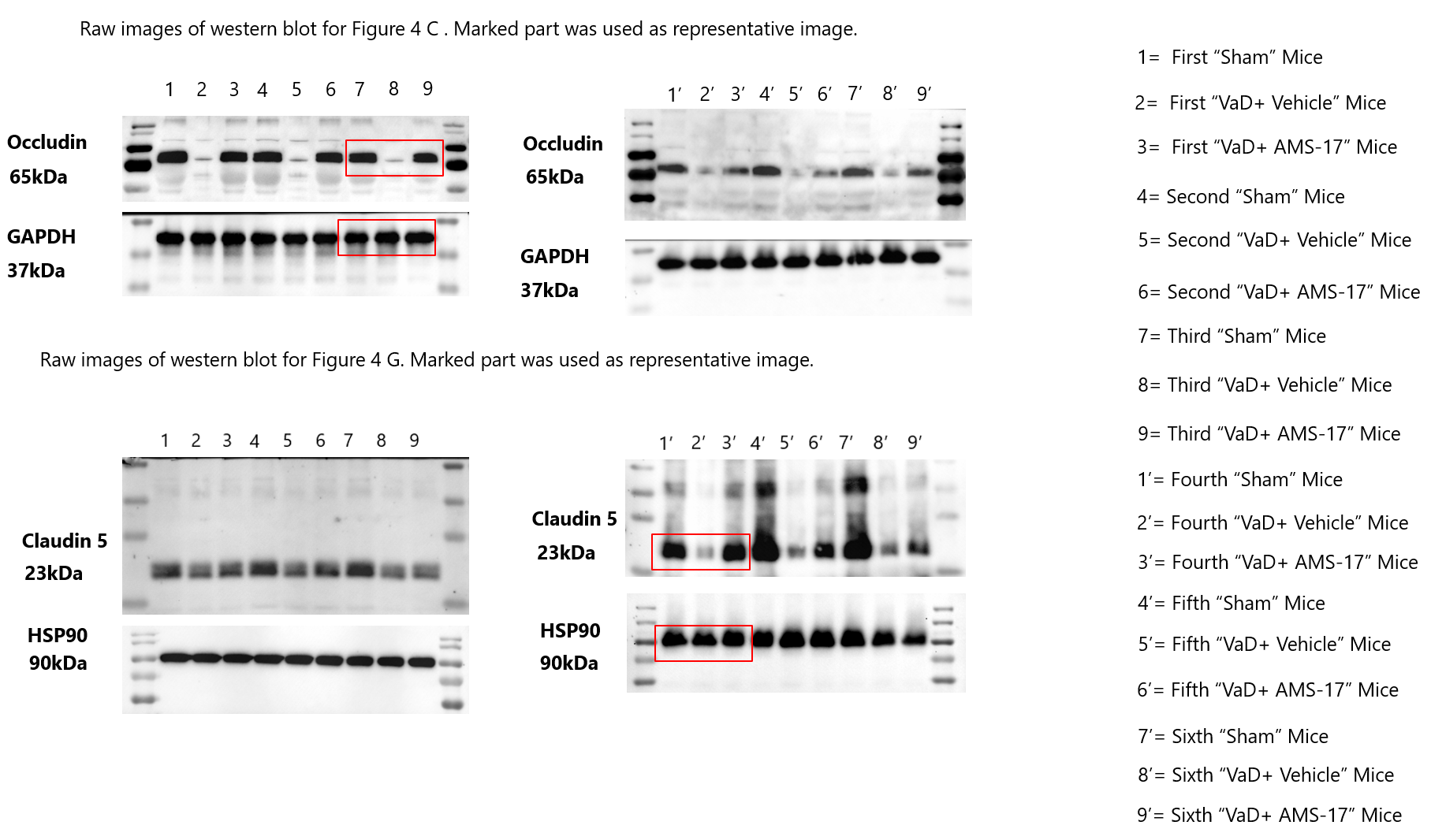


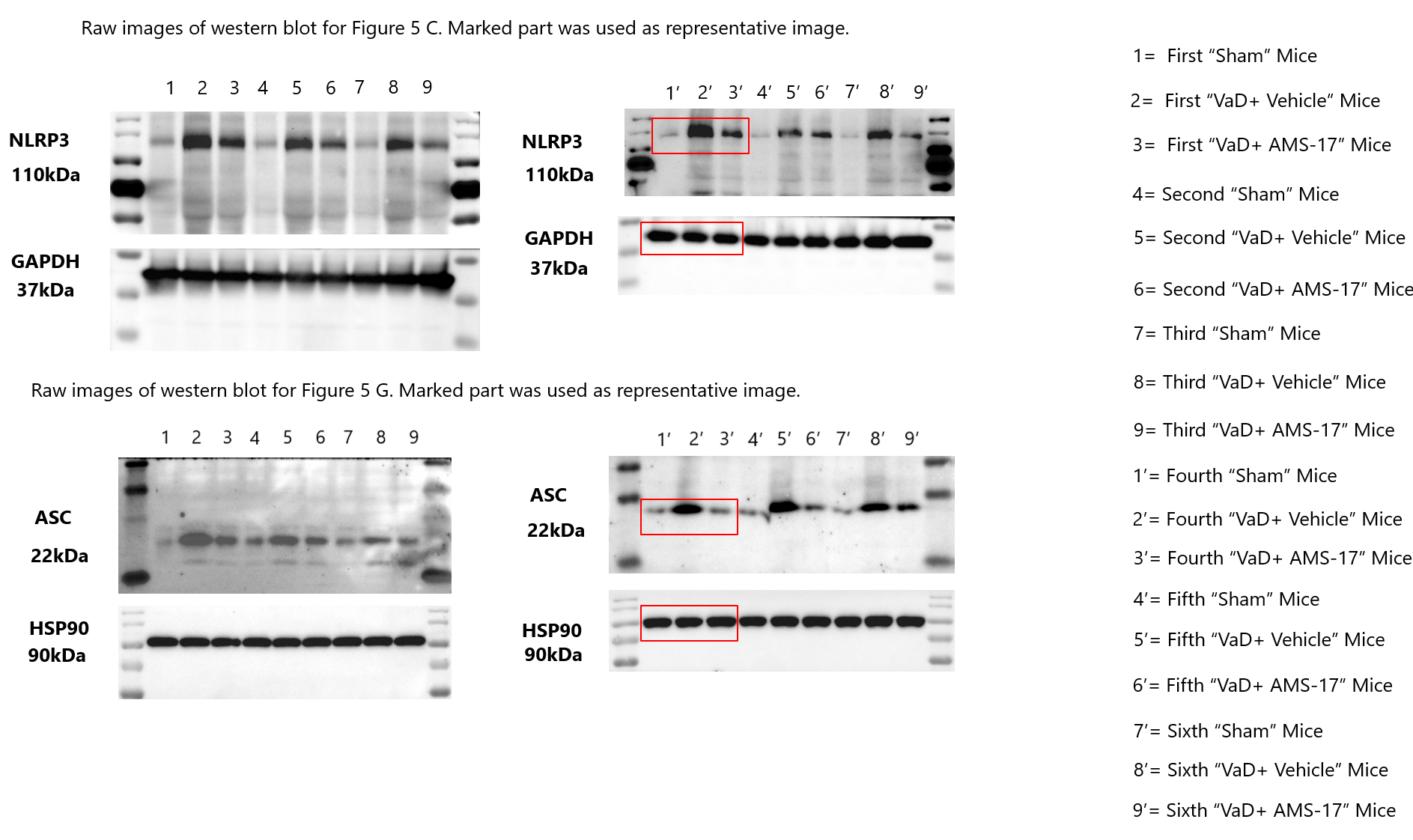


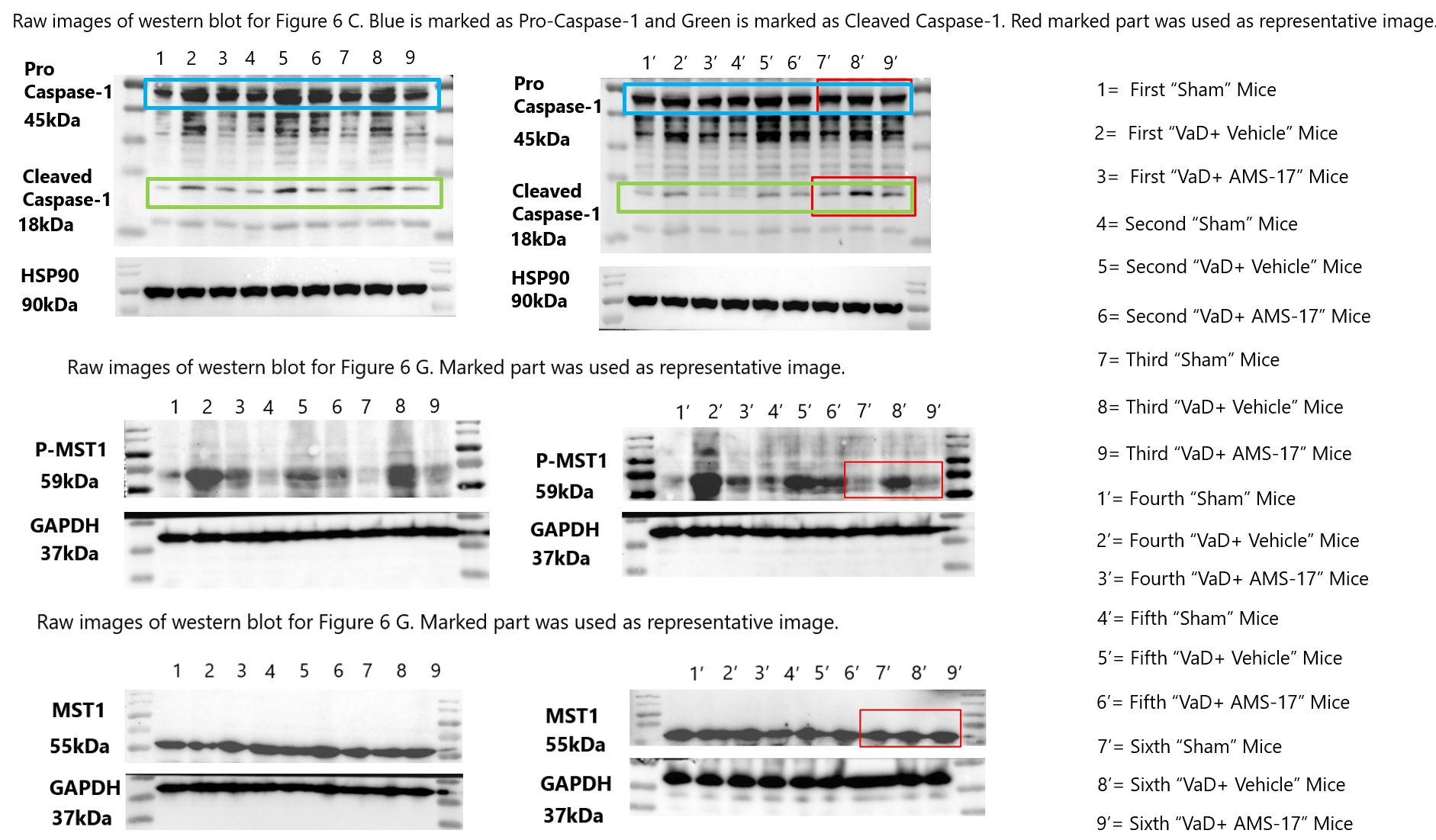

Supplement: Supplementary file 1 — Supplementary Material 1 [file 12987_2025_665_MOESM1_ESM.docx]
